# RNA as a component of scrapie fibrils

**DOI:** 10.1038/s41598-024-55278-0

**Published:** 2024-02-29

**Authors:** Leslie R. Bridges

**Affiliations:** 1 Neuropathology, Cellular Pathology, South West London Pathology, St George’s Hospital, St George’s University Hospitals NHS Foundation Trust, London, UK; 2 Molecular and Clinical Sciences Research Institute, St George’s University of London, London, UK

**Keywords:** Diseases of the nervous system, Neurodegeneration, Neuroscience, Structural biology, Electron microscopy, Cryoelectron microscopy, Neurology, Neurological disorders, Prion diseases

## Abstract

Recently, electron cryo-microscopy (cryo-EM) maps of fibrils from the brains of mice and hamsters with five infectious scrapie strains have been published and deposited in the electron microscopy data bank (EMDB). As noted by the primary authors, the fibrils contain a second component other than protein. The aim of the present study was to identify the nature of this second component in the published maps using an in silico approach. Extra densities (EDs) containing this component were continuous, straight, axial, at right angles to protein rungs and within hydrogen-bonding distance of protein, consistent with a structural role. EDs co-located with strips of basic residues, notably lysines, and formed a conspicuous cladding over parts of the N-terminal lobe of the protein. A Y-shaped polymer consistent with RNA was found, in places forming a single chain and at one location forming a duplex, comprising two antiparallel chains, and raising the intriguing possibility of replicative behaviour. To reflect the monotonous nature of the protein interface, it is suggested that the RNA may be a short tandem repeat. Fibrils from brains of patients with Alzheimer’s disease, Parkinson’s disease, amyotrophic lateral sclerosis and other neurodegenerations also contain EDs and may be of a similar aetiology.

## Introduction

One of the pedestals of the protein-only prion hypothesis is the idea that scrapie strains are enciphered by protein conformations, rather than nucleic acids^1,2^. On the other hand, it has recently been affirmed that cofactors (including RNA) are essential strain determinants^3^. High resolution structural models of scrapie fibrils have therefore been eagerly anticipated, to contribute to this debate. Near-atomic level cryo-EM maps and corresponding protein structures of five scrapie strains^4–8^ have recently been deposited in the EMDB^9^ and protein data bank (PDB)^10^. Whilst there are certainly differences between the protein conformations, there is also a conspicuous second component, within EDs, coordinating with lysines and other basic residues. Lysine-coordinating EDs are a common feature of fibrils from the brains of patients with AD and other neurodegenerations and evidence suggests that they contain RNA^11^. The aim of the present study was to identify the constituent molecule(s) of EDs in scrapie fibrils, by in silico methods, using cryo-EM and atomic data from the five scrapie strains^4–8^, from the EMDB^9^ and PDB^10^ public repositories. The results are discussed in the context of the on-going debate about whether scrapie is due to an protein-only prion or a virus-like agent.

### The nature of EDs

**Figure 1:**
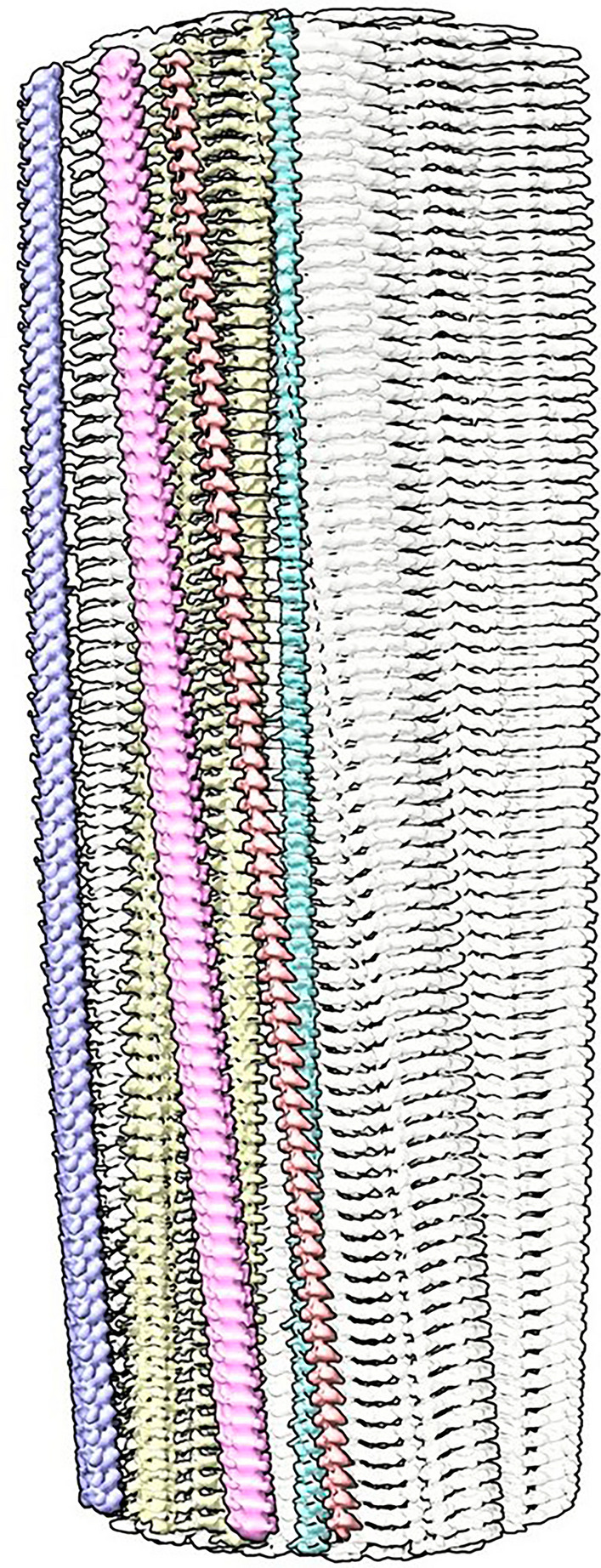
**Supported stack.** PrP fibril of a22L mouse scrapie. Extra densities (colours) are upright, at right angles to protein rungs (white, transparent), consistent with a structural role as guide and support. Distance between protein rungs is 4.8 Å. Image of EMDB 28089^7^ created with UCSF ChimeraX^22^.

**Figure 2:**
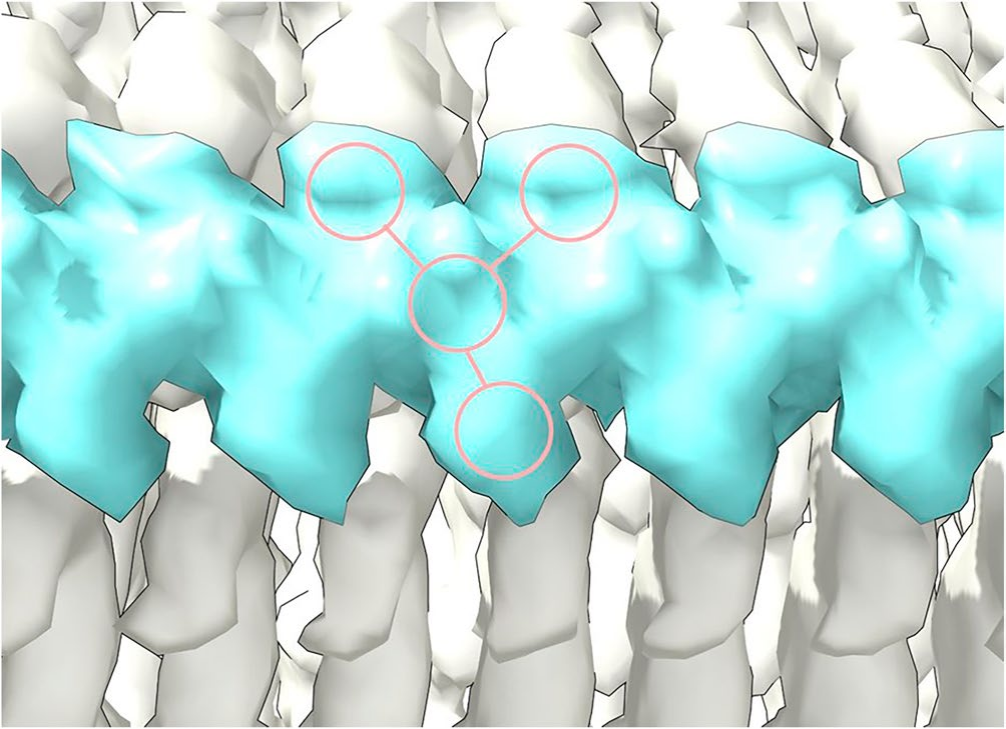
**Straight polymer.** Extra density (ED) 28089-109 from PrP fibril of a22L mouse scrapie. Continuous density and repeating features are consistent with a polymer. Note 3-blob pattern and Y-shaped connectivity. Image of EMDB 28089^7^ created with UCSF ChimeraX^22^.

Extra densities (EDs) are present in scrapie fibrils (Fig. 1), similar to those in Alzheimer’s disease (AD) and other human neurodegenerations^11^. They are continuous with a repeat distance matching that of protein (about 4.8 Å) (Fig. 2). They have well-defined features, a 3-blob morphology and Y-shaped connectivity. They are within hydrogen bonding distance of protein and maintain a constant attitude to the protein, twisting and arcing gently with the fibril. They are orthogonal, in the dual sense that they are straight and run at right angles to the protein.

The arrangement suggests a supported stack (Fig. 1), in which near-planar protein monomers form a stack, supported by uprights composed of the second component. This novel structure, also seen in AD and other human neurodegenerations^11^, is seemingly unique in nature and a hallmark of these diseases.

### The protein environment

**Figure 3:**
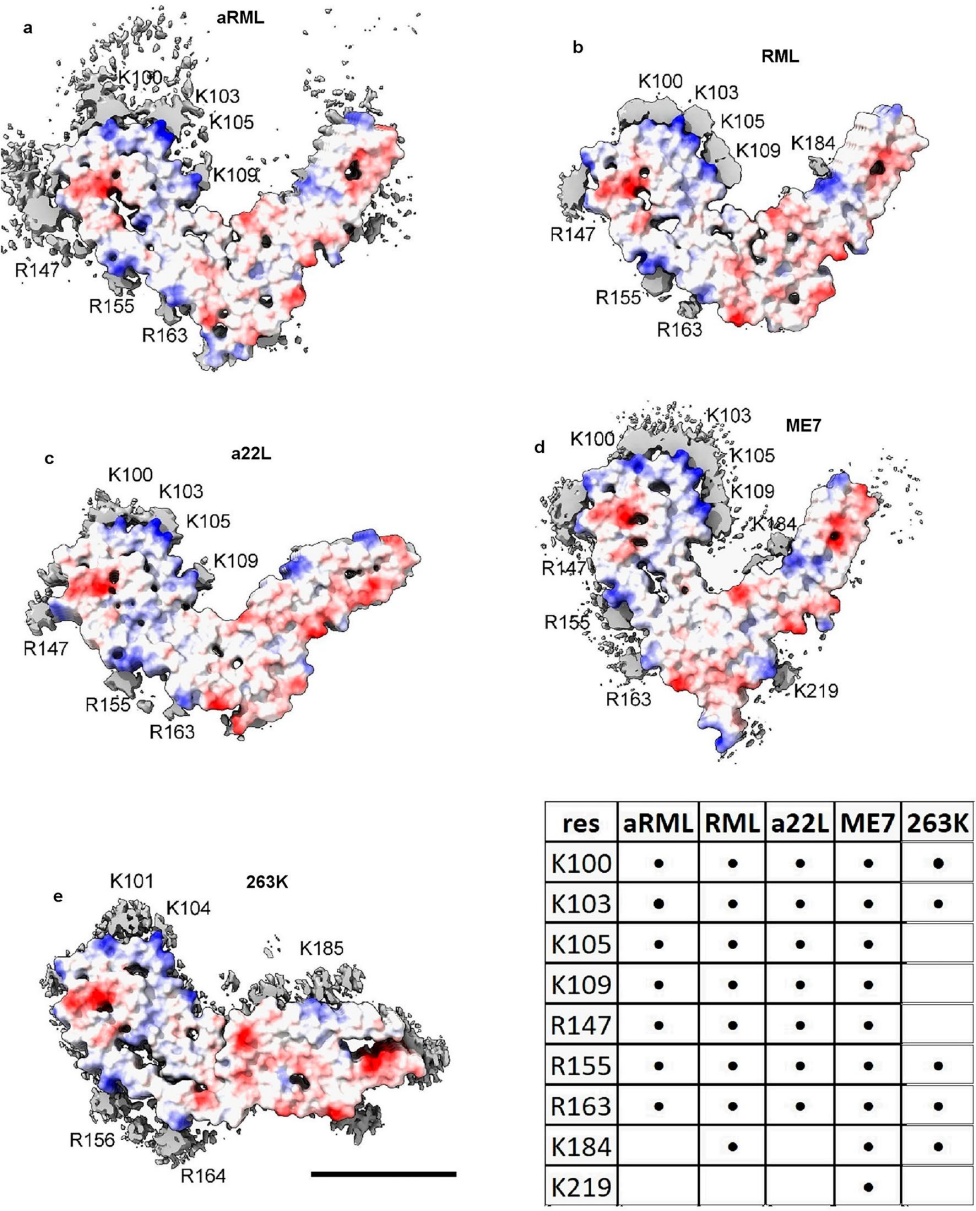
**Electrostatic potential surfaces and EDs.** Locations of EDs in PrP fibrils from five scrapie strains as summarised in the table. Note lysine patch (K100, K103, K105 and K109) and arginine patch (R147, R155 and R163) on medial and lateral aspects of N-terminal lobe. Electrostatic potential surfaces of proteins are shown, electropositive blue and electronegative red. Images of EMDB 25824 and PDB 7td6 in aRML mouse scrapie^6^ (**a**), 13989 and 7qig in RML mouse scrapie^5^ (**b**), 28089 and 8efu in a22L mouse scrapie^7^ (**c**), 15043 and 8a00 in ME7 mouse scrapie^8^ (**d**) and 23459 and 7lna in 263K hamster scrapie^4^, scale bar 50 Å (**e**), created with UCSF ChimeraX^22^.

As in AD and other human neurodegenerations^11^, EDs in scrapie coordinate with lysines and other polar residues. EDs in scrapie (Fig. 3) coordinate with lysine motifs 100**K**P**SK**103, 103**K**P**K**105, 105**K**T**N**L**K**109 and 109**KH**110 (mouse numbering, residues facing ED in bold). EDs also coordinate with arginines R147, R155 and R163. In some strains, there are contacts with T94, Q159 and Y161 and a floating ED between arginines R155 and R163. In the C-terminal lobe, EDs are found at 184**K**Q**H**186 and 217**Y**Q**K**219 in some strains.

The lysine and arginine patches (K100-K109 and R147-R163) fall within regions highlighted as important in previous research. Residues 90–112 are involved in the conformational transition from PrP^C^ to PrP^Sc^ (refs.^2,12^). The central lysine cluster (K100-K109) is predicted to bind charge-neutralising cofactors^13^. Two domains, 91–115 and 144–163, are relatively solvent-protected in infectious (cofactor) PrP^Sc^ compared to non-infectious (protein-only) PrP^Sc^ (ref.^14^).

**Figure 4:**
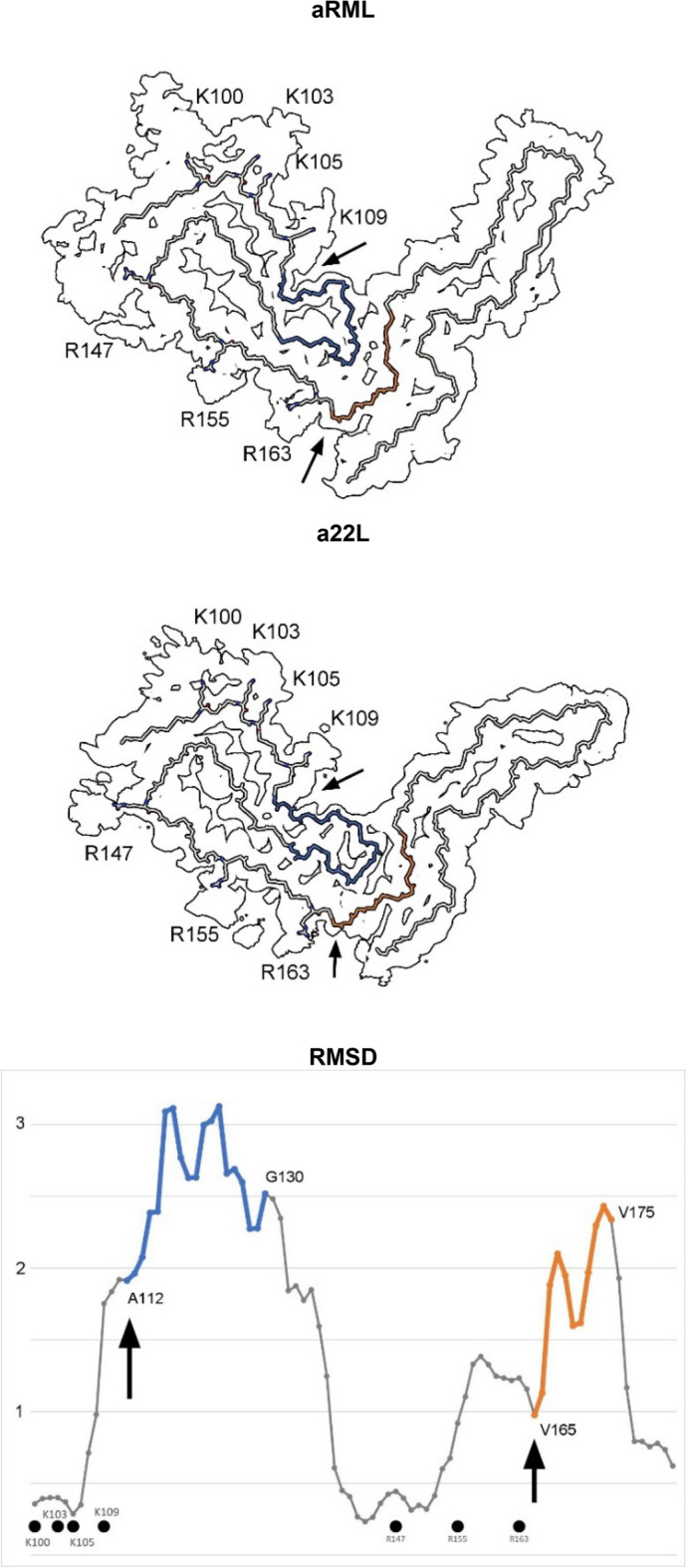
**Comparison of scrapie strains.** The RMSD chart shows peak variability (commencing at arrows) immediately downstream of the lysine and arginine patches. The atomic structures (colours correspond to chart) indicate articulation at the inter-lobar interface, consistent with an allosteric effect of ligands within EDs at K100, K103, K105, K109, R147, R155 and R163 in syngeneic mice. Note difference in angle between N- and C-terminal lobes. Images of EMDB 25824 and PDB 7td6 in aRML^6^ (**a**) and 28089 and 8efu in a22L mouse scrapie^7^ (**b**) created with UCSF ChimeraX^22^.

Protein monomers are boomerang-shaped with distinctive angles between lobes (Figs. 3, 4). This angle is determined by variable regions, namely the head of the N arch (residues 112–130) and the central strand (residues 165–175), forming the inter-lobar interface^7,8^.

This is reflected in the root mean square deviation (RMSD) plot comparing a22L and aRML mouse scrapie strains (Fig. 4). Since the mice are syngeneic, the differences are unrelated to amino acid sequence. On the other hand, both variable regions are immediately downstream of EDs, suggesting an effect of ligands on protein conformation.

Overall, the evidence suggests that EDs contain a linear polyanion which binds to cationic strips on the surface of the protein and exerts a tethering effect on protein conformation.

### The constituent molecule

**Figure 5:**
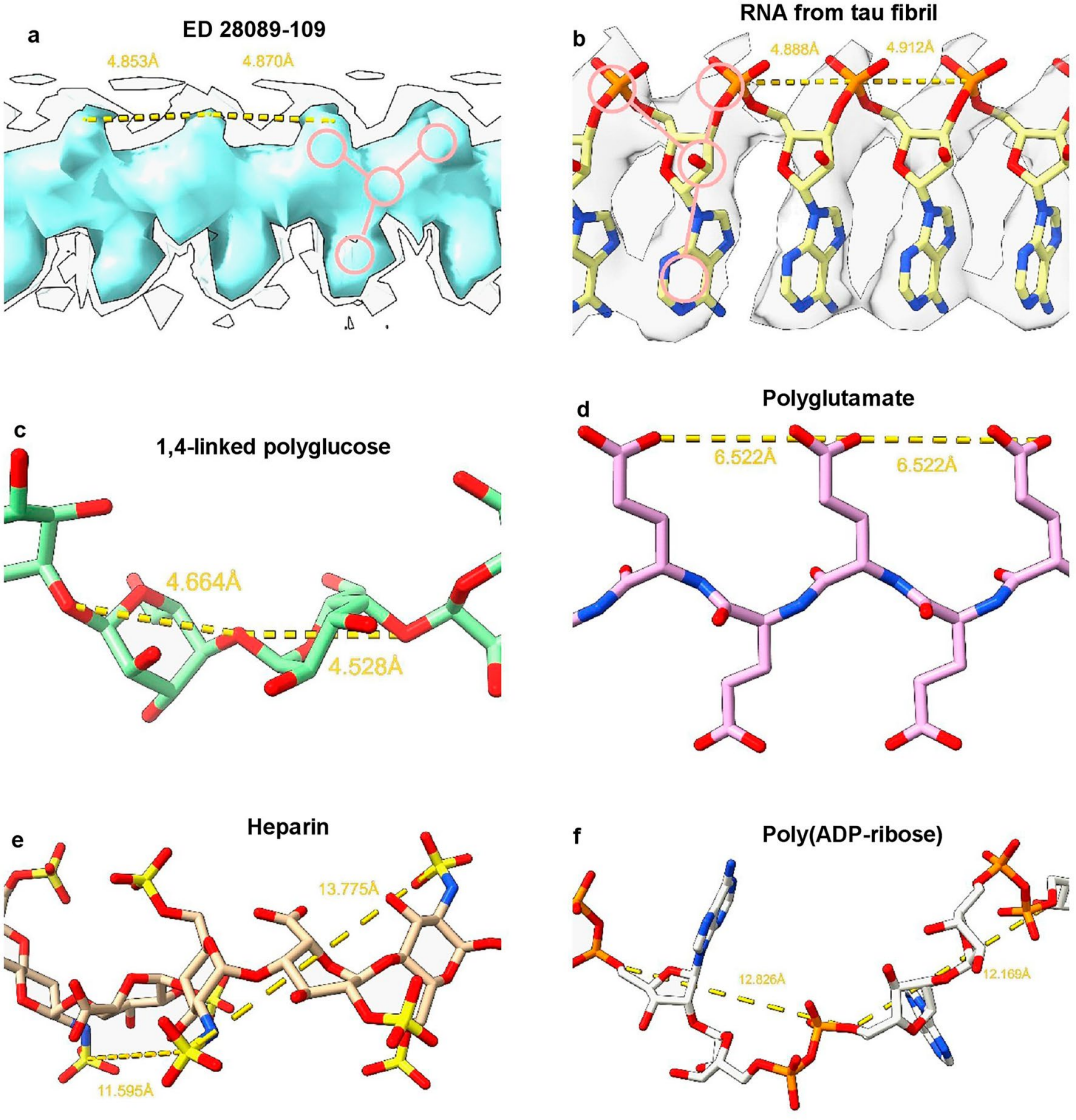
**Candidate molecules.** (**a**) ED 28089-109 from PrP fibril of a22L mouse scrapie is straight with a 3-blob pattern and Y-shaped connectivity. (**b**) ED from in vitro tau fibril, known by experiment to contain a straight form of RNA. Note 3-blob pattern and Y-shaped connectivity. (**c**) 1,4-linked polyglucose is fairly straight with a repeat distance close to that of protein but is not polyanionic and has a single-blob pattern. (**d**) Polyglutamate is a straight polyanion but is multidentate with a repeat distance different to protein. (**e**) Heparin is a coiled, multidentate polyanion with a long repeat distance. (**f**) Poly (ADP-ribose) is a coiled, multidentate polyanion with a long repeat distance. Images of (**a**) EMDB 28089^7^, (**b**) EMDB 25364 and PDB 7sp1^15^, (**c**) Pubchem^37^ 90478052 rebuilt in UCSF ChimeraX^22^, (**d**) polyglutamate built as beta-strand in UCSF ChimeraX^22^, (**e**) PDB 3iri^38^ and (**f**) PDB 4l2h^39^ rebuilt in UCSF ChimeraX^22^, created with UCSF ChimeraX^22^.

Considering the fibrils from the Rocky Mountain (RM) lab^4,6,7^, their authors state that EDs may contain non-protein ligands. As exemplar, ED 28089-109 (Fig. 5a) is straight (angle 179.5°) with a repeat distance about 4.8 Å matching that of protein. It has a 3-blob morphology and Y-shaped connectivity and its protein environment suggests a polyanion.

A straight form of RNA has been described, complexed to tau fibrils^15^ (Fig. 5b). Tau protein was incubated with RNA and formed fibrils in which RNA is straight (angle 179.1°) with a repeat distance about 4.8 Å matching that of protein. It runs parallel to the fibril axis, at right angles to protein rungs and within hydrogen bonding distance of basic residues. The morphology is 3-blob (corresponding to phosphate, ribose and base) and the connectivity is Y-shaped.

Interestingly, Watson and Crick^16^ postulated the existence of a straight form of nucleic acid. Furthermore, straight RNA (named ortho-RNA, oRNA) was modelled, in silico^11^, into an ED coordinating with K43 and K45 in an alpha-synuclein fibril from multiple system atrophy (MSA)^17^.

1,4-linked polyglucose (Fig. 5c), a glycogen-like molecule, is known to form a molecular scaffold in scrapie fibrils^18^, and is rather straight (angle 159.2°) with a repeat distance of 4.6 Å, close to the that of protein. On the other hand, it is single-blob rather than 3-blob, not Y-shaped and not a polyanion.

Polyglutamate (Fig. 5d), as beta-strand, is a straight polyanion (angle 179.7°), but multidentate rather than Y-shaped, with a repeat distance of 6.6 Å, out of step with protein, and unlikely to be available to brain cells.

Heparin (Fig. 5e) is polyanionic but coiled rather than straight (angle 91.1°) with a repeat distance of 12.7 Å out of step with protein. Heparan sulfate is similar^19^, and is available on the surface of many cell types.

Poly (ADP-ribose) (Fig. 5f) is a potentially bioavailable polyanion but coiled (angle 126.9°) with a repeat distance of 12.5 Å.

**Figure 6:**
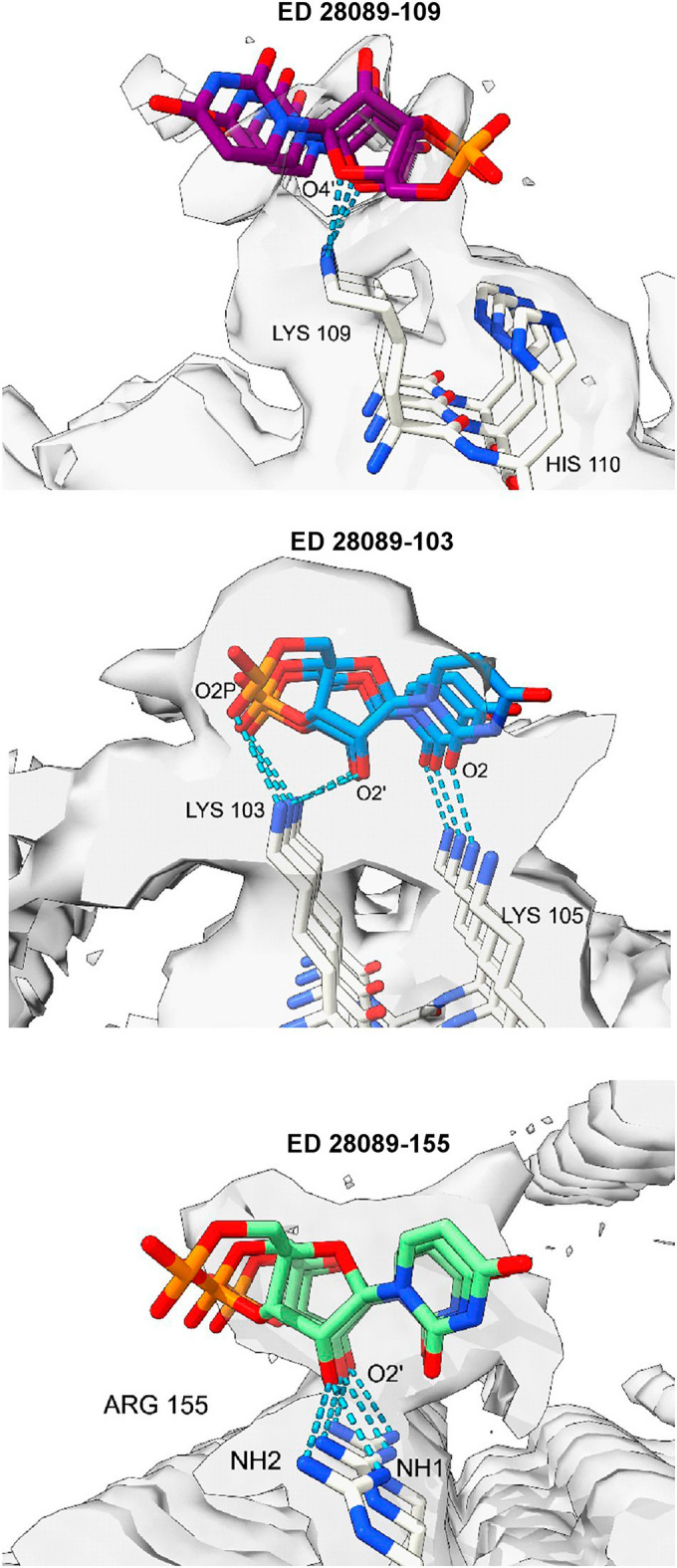
**RNA is a feasible ligand for PrP.** Ortho-RNA forms a rich symmetrical network of hydrogen bonds with PrP, consistent with a role in determining protein conformation. Images of EMDB 28089 and PDB 8efu^7^ created with UCSF ChimeraX^22^, oRNA modelled in silico as described^11^, oRNA poses generated with AutoDock Vina^20^.

Molecular docking shows feasible poses for oRNA with lysines and arginines, with good AutoDock Vina^20^ scores and MolProbity^21^ clashscores (Fig. 6). The docked poses overlap with EDs and form a rich symmetrical network of hydrogen bonds.

**Figure 7:**
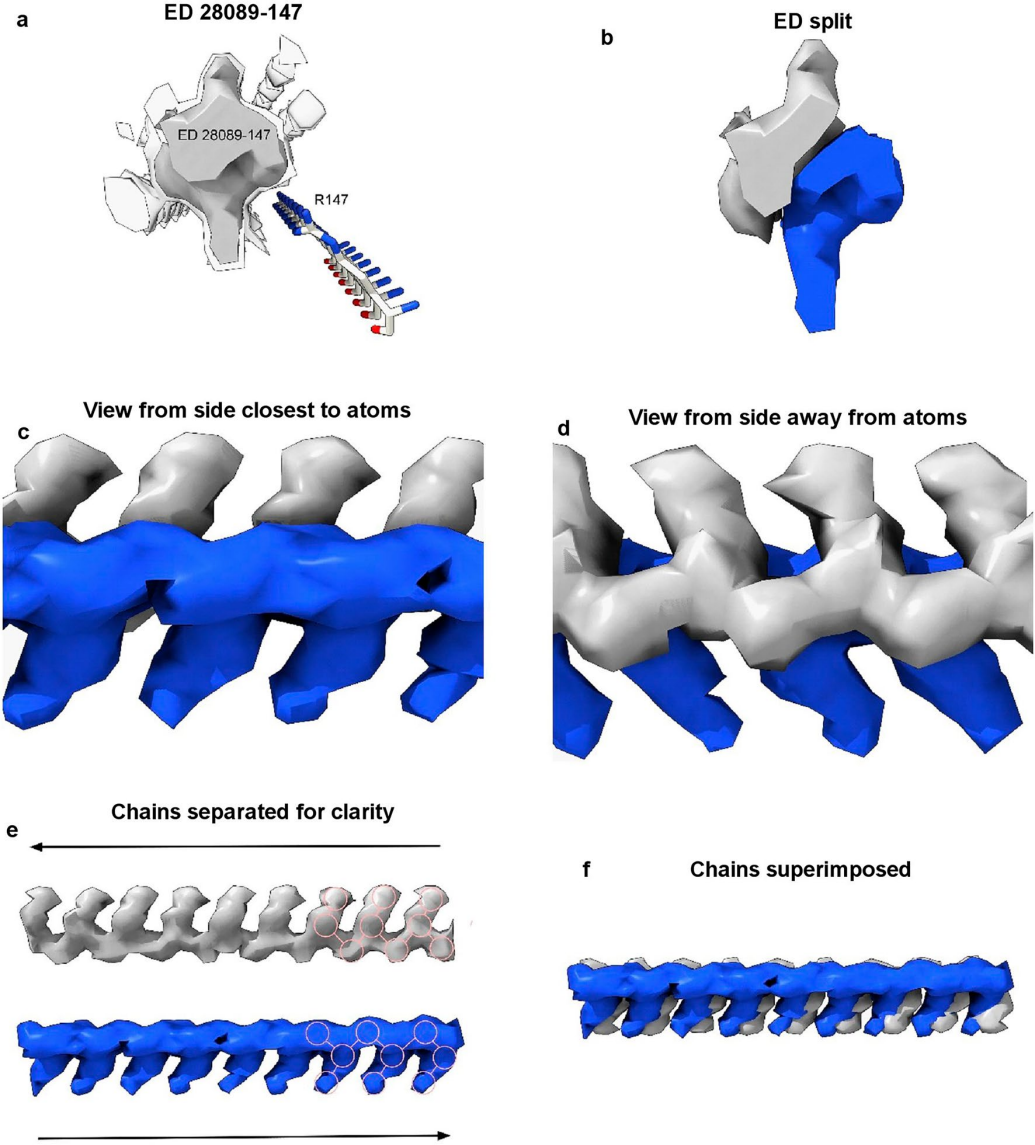
**Duplex antiparallel chains.** (**a**) ED 28089-147 from PrP fibril of a22L mouse scrapie, at authors’ contour level (transparent) and increased level (opaque). (**b**) ED is split by proximity to terminal nitrogens of R147. Note symmetry. (**c**) and (**d**) Side views, indicating two near-identical chains. (**e**) The chains have been separated. Both have a 3-blob pattern and Y-shaped connectivity consistent with RNA. Their directions are opposite (antiparallel). (**f**) The superimposed chains are highly correlated. Images of EMDB 28089 and PDB 8efu^7^ created with UCSF ChimeraX^22^.

One ED (28089-147), after splitting by proximity to R147 terminal nitrogens, shows two anti-parallel chains arranged as a duplex (Fig. 7). The two chains have 3-blob morphology and Y-shaped connectivity and run in opposite directions. They are similar in appearance, and after a 180° rotation placing them in the same direction, can be fitted one into the other (fit in map correlation 0.893 in USCF ChimeraX^22^).

**Figure 8:**
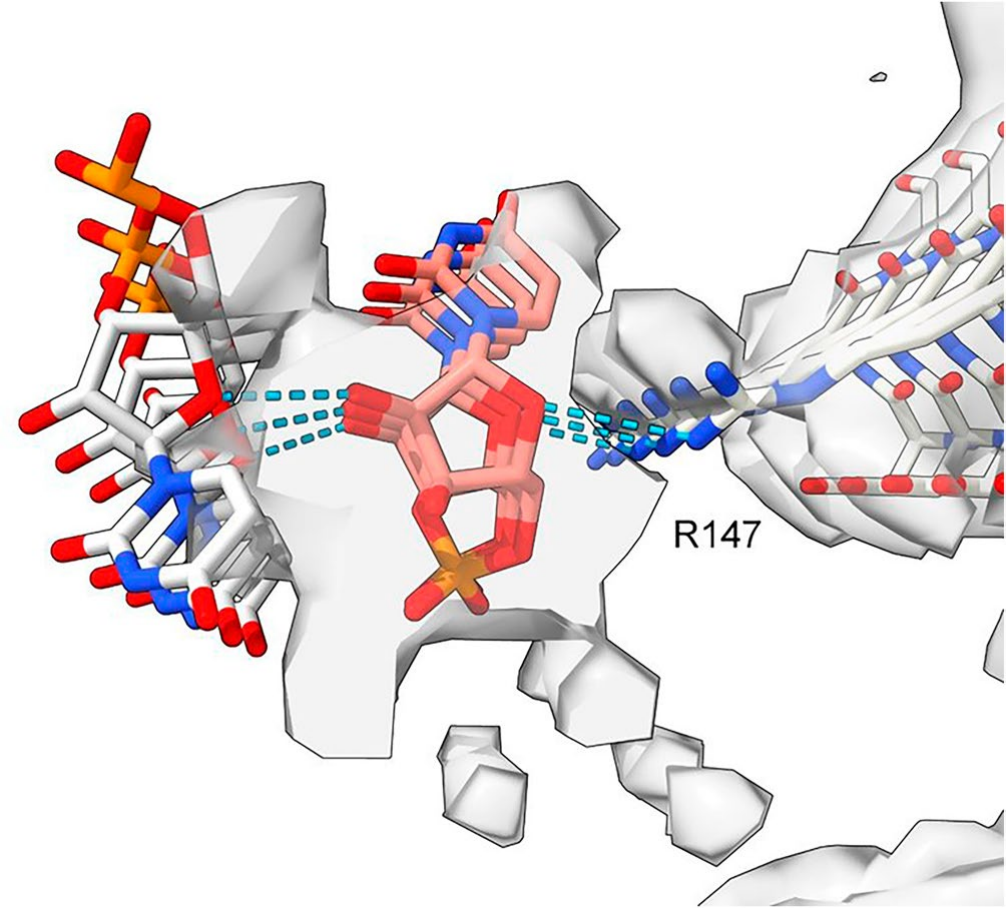
**Model of duplex RNA.** ED 28089-147 from PrP fibril of a22L mouse scrapie. A duplex form of ortho-RNA is docked to protein; good geometry, symmetrical hydrogen bonds and a reasonable fit to the ED are found. The oRNA chains are anti-parallel and congruent with the split ED (see Fig, 7), raising the possibility of replicating RNA. Image of EMDB 28089 and PDB 8efu^7^ created with UCSF ChimeraX^22^, duplex oRNA modelled in silico as described^11^, oRNA poses generated with AutoDock Vina^20^.

Furthermore, an atomic model of duplex oRNA was posed opposite R147 using molecular docking (Fig. 8). The resulting model has symmetrical hydrogen bonds, good geometry and a reasonable fit to the ED (fit in map correlation 0.767 in USCF ChimeraX^22^). This indicates that duplex RNA is a feasible constituent of the ED. Whilst the duplex structure does not have the structure of double-stranded RNA (dsRNA), it could be a refolded form of dsRNA.

Estimates of molecular weight (MW) were carried out on EDs in the PrP fibril of a22L mouse scrapie. MW per unit of ED were 216 Daltons (Da) for ED 28089-109, 350 Da for ED 28089-147, 389 Da for ED 28089-155 and 94 Da for ED 28089-163. The fused mass of ED overlying K100 to K105 had a MW of 1029 Da. Such estimations are indeterminate because the occupancy of the constituent molecules is unknown, but EDs at K109 and R163 are consistent with single RNA strands at 67% and 26% occupancies respectively. EDs at R147 and R155 are consistent with duplex RNA at 55% and 61% occupancies respectively. The conglomerate ED between K103 and K105 has a MW equivalent to 6 RNA strands at 54% occupancy.

Other evidence also points to RNA. RNA is complexed to PrP in amyloid plaques in scrapie^23^. EM micrographs of scrapie fibrils show tails, sensitive to Zn^2+^ ions, suggestive of RNA^24^. RNA is known to promote PrP^Sc^ formation in vitro^25^.

**Figure 9:**
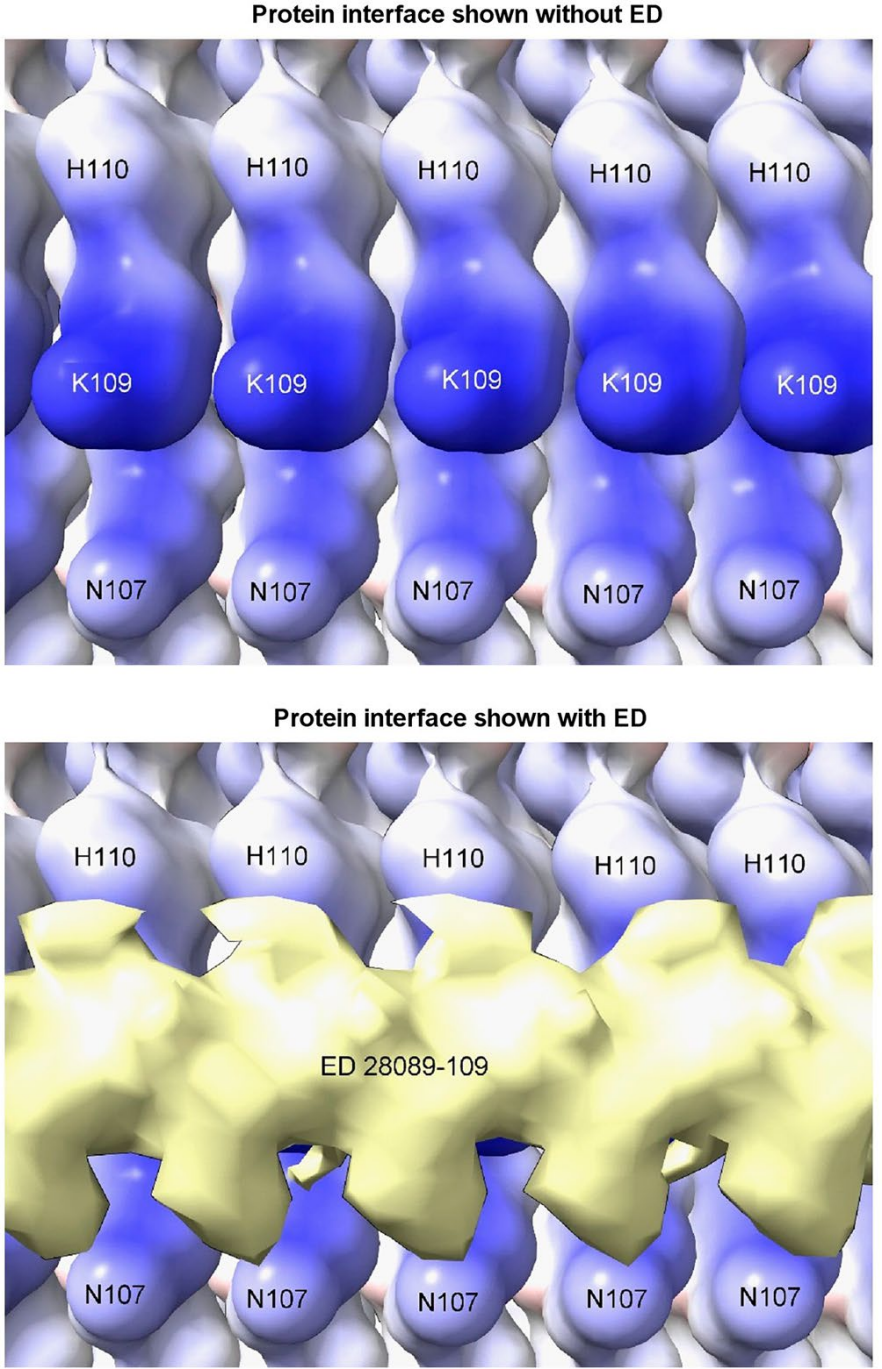
**Repetitive interface.** PrP fibril from a22L mouse scrapie shown with and without overlying ED. The protein interface is repetitive, suggesting that the constituent molecule of the ED may also be repetitive (e.g. a homopolymer or short tandem repeat of RNA). Electrostatic potential surface of protein is shown, electropositive blue and electronegative red. Images of EMDB 28089 and PDB 8efu^7^ created with UCSF ChimeraX^22^.

Due to averaging, it is not possible to read the base sequences in the present studies. However, given that the protein interface is identical at every rung (Fig. 9), for consistent binding the RNA might also be repetitive, either as a homopolymer or short tandem repeat. Host-like repetitive nucleic acid^26^ and single-stranded DNA (ssDNA) with the palindromic sequence (TACGTA)_n_^27^ were found in hamster scrapie. Molecular docking studies show that ssDNA and RNA are potentially interchangeable^11^.

Considering the fibrils from the University College London (UCL) lab^5,8^, the authors assert that EDs represent phosphotungstic acid (PTA), a polyanionic reagent used in the preparation of their fibrils and not used by the RM lab. The regular appearances are consistent with this idea. However, the presence of EDs in fibrils from both labs suggests that natural ligands are usually present and might therefore have been displaced in the UCL fibrils.

### Comparison with human neurodegenerations

Evidence here suggests that scrapie and human neurodegenerative diseases are part of a spectrum, differing in degree but not in kind, and that RNA is a unifying factor.

Features in common include slow tempo and cell-to-cell spread within the brain. Experimentally, they can usually be transmitted to other organisms, although natural transmission is probably confined to certain PrP diseases (notably kuru and scrapie).

**Figure 10:**
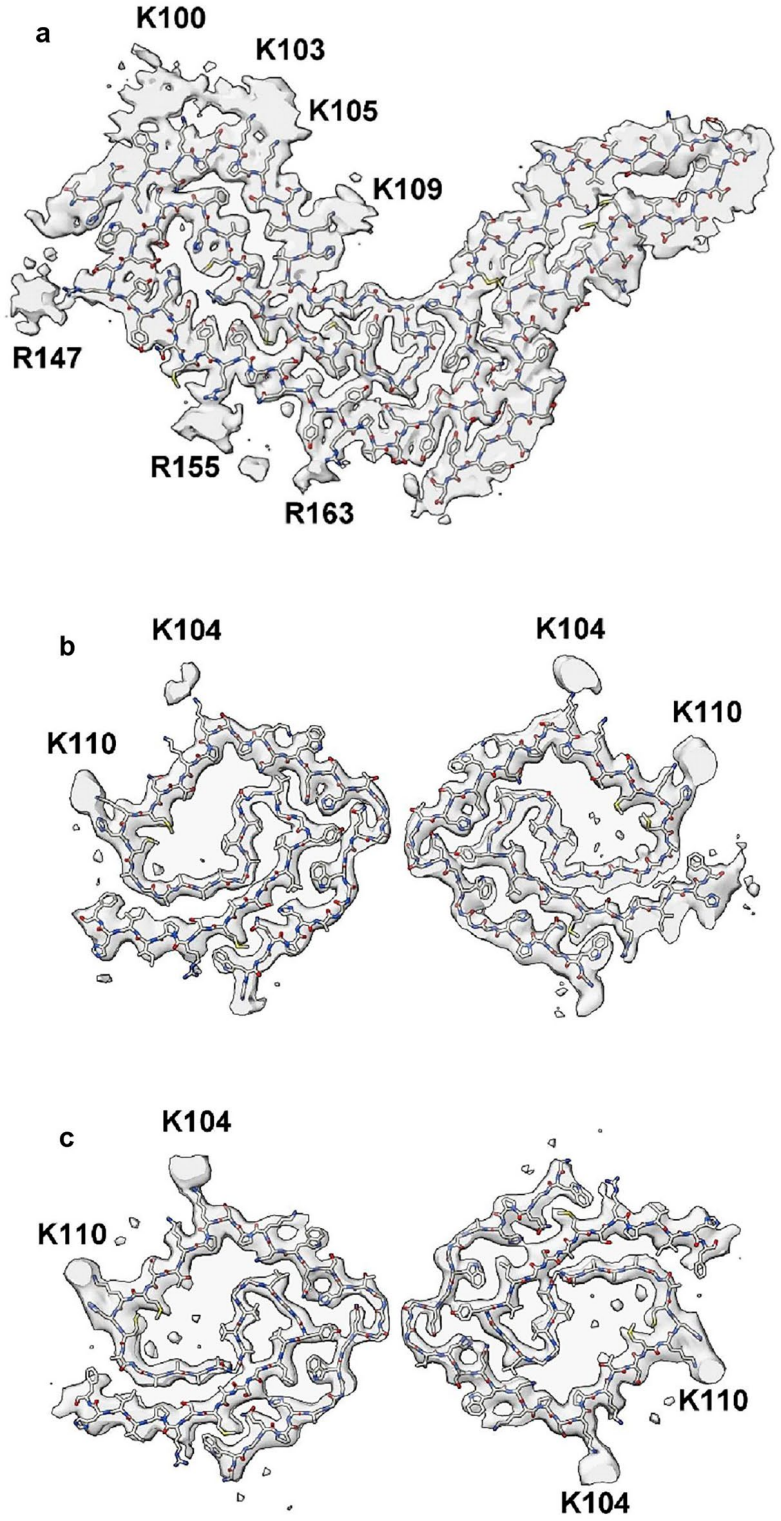
**Comparison of scrapie and GSS.** PrP fibrils from a22L mouse scrapie (**a**) versus human GSS (**b**, **c**). The scrapie fibril is a single large protofilament whereas the GSS fibrils have two smaller protofilaments. The burden of EDs is greater in scrapie than GSS. Images of EMDB 28089 and PDB 8efu^7^ (**a**), 26613 and 7un5^28^ (**b**) and 26607 and 7umq^28^ (**c**) created with UCSF ChimeraX^22^.

**Figure 11:**
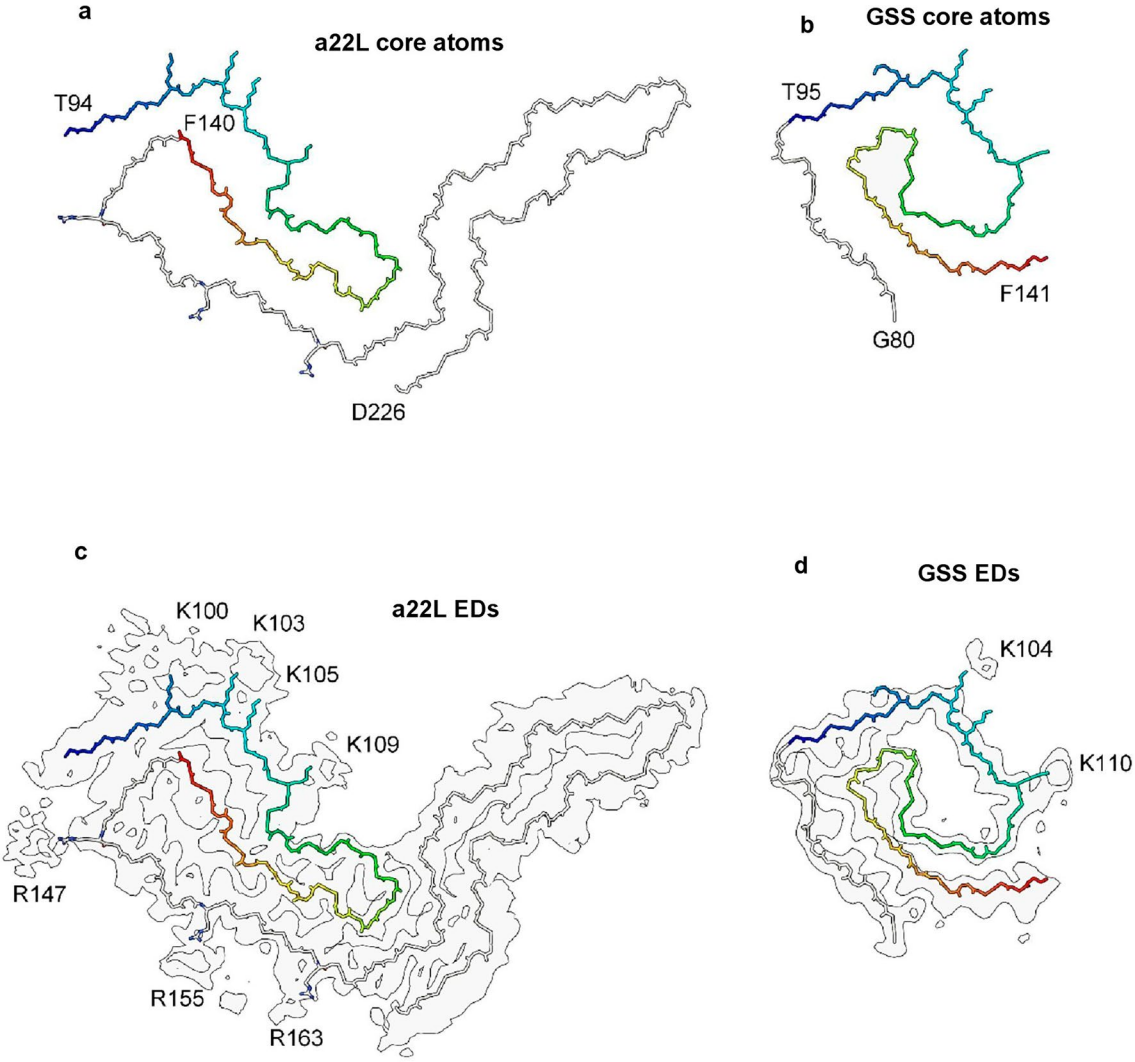
**GSS lacks arginine patch.** PrP fibrils from a22L mouse scrapie (**a**, **c**) and the human neurodegenerative disease GSS (**b**, **d**). Although the lysine patch is present in both, there are EDs at K100, K103, K105 and K109 in scrapie but only at K104 and K110 in GSS. The arginine patch (with EDs present at R147, R155 and R163 in scrapie) is absent in GSS, due to truncation of the protein core. Images of EMDB 28089 and PDB 8efu^7^ (**a**, **c**) and 26613 and 7un5^28^ (**b**, **d**) created with UCSF ChimeraX^22^.

Gerstmann-Sträussler-Scheinker disease (GSS) fibrils have two protofilaments whereas scrapie typically only has one (Fig. 10). However, core size is smaller in GSS than in scrapie (Fig. 11). Whilst the lysine patch (K100–K109) is present in both, EDs are fewer, smaller and less closely-packed in GSS. Furthermore, the arginine patch (R147–R163), known to be associated with infectivity in scrapie^14^, is absent in GSS. The F198S form of GSS^28^ has a slow tempo and little or no transmissibility. Furthermore, it has a conspicuous co-pathology, in the form of tau paired helical filament (PHF) and straight filament (SF) fibrils. Arguably, it resembles AD more than scrapie or Creutzfeldt-Jakob disease (CJD), despite being a disease of PrP.

The core sizes of the five scrapie strains range from 132 to 138 residues. In comparison, in human neurodegenerations, core sizes range from 34 residues (beta-amyloid fibril in AD, PDB 7q4b^29^) to 135 residues (TMEM106B fibril in AD, PDB 7qvc^30^). Protofilament sizes of 94 residues (tau fibril in Pick’s disease, PDB 6gx5^31^), 110 residues (tau fibril in progressive supranuclear palsy (PSP), PDB 7p65^32^) and 115 residues (tau fibril in argyrophilic grain disease (AGD), PDB 7p6d^32^) are only somewhat smaller than scrapie. Thus, whilst core sizes are generally smaller in human neurodegenerations compared to scrapie, this is not an absolute distinction. Furthermore, where protofilaments are typically paired, the combined fibril size in terms of residues is, in some instances even larger than scrapie, such as 146 residues (tau PHF fibril in AD, PDB 5o3l^33^). Since the protofibrils in GSS are also paired, the combined residue count is 124 residues (PrP fibrils in GSS, PDB 7umq and PDB 7un5^28^), close to that of scrapie. It seems, therefore, that there is no reason why the core size per se should determine infectivity. More likely, the possession of key motifs including lysines is of greatest importance.

**Figure 12:**
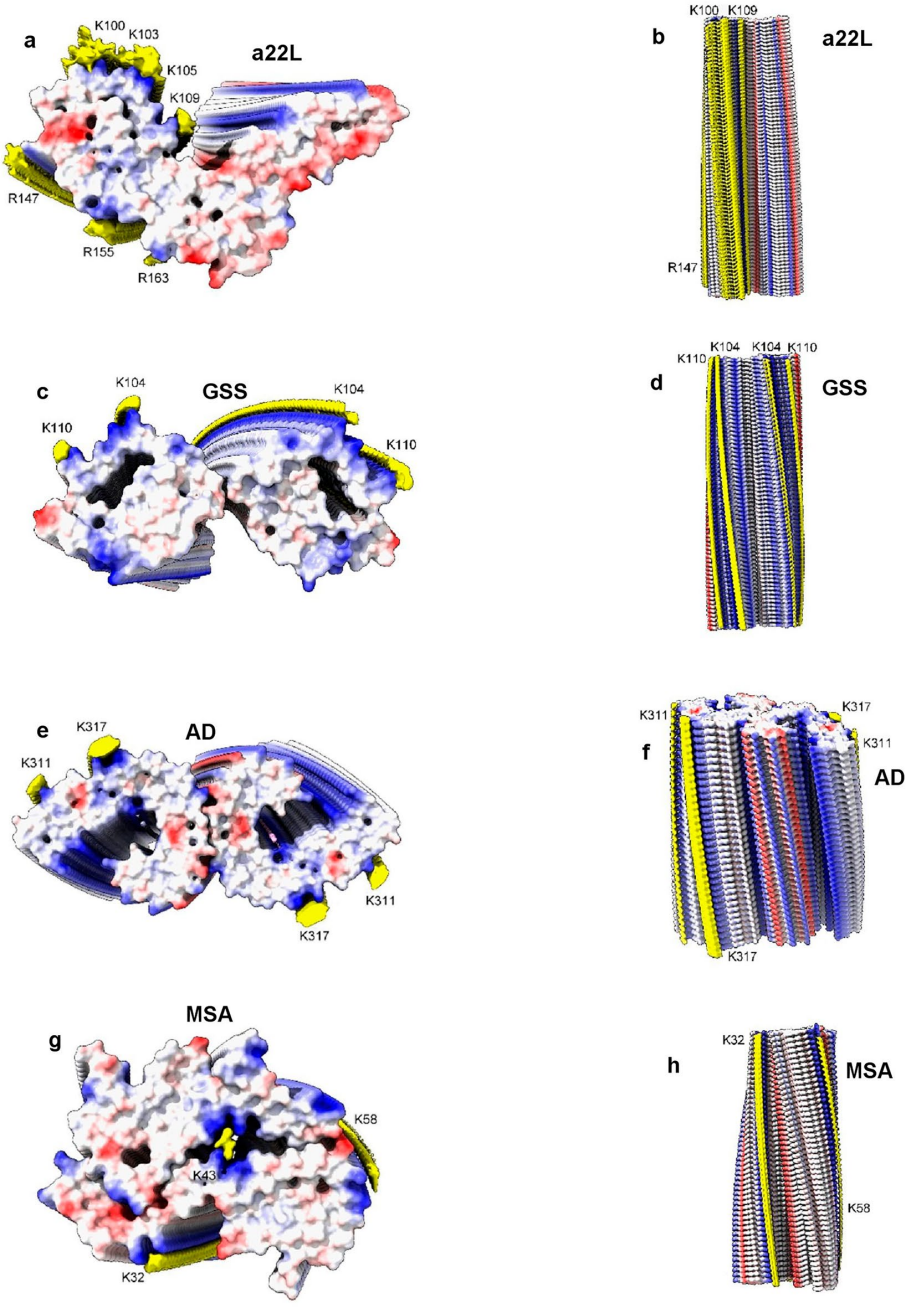
**Scrapie and human neurodegenerations.** PrP fibrils from a22L mouse scrapie (**a**, **b**) and GSS (**c**, **d**), tau PHF fibril from AD (**e**, **f**) and alpha-synuclein fibril from MSA (**g**, **h**). Electrostatic potential surfaces of proteins are shown, electropositive blue and electronegative red. EDs are shown in yellow. Notwithstanding differences in core size and size and packing density of EDs, the fibrils are more similar than different. Distance between protein rungs is about 4.8 Å. Images of EMDB 28089 and PDB 8efu^7^ (**a**, **b**), 26613 and 7un5^28^ (**c**, **d**), 26663 and 7upe^34^ (**e**, **f**) and 10650 and 6xyo^17^ (**g**, **h**) created with UCSF ChimeraX^22^.

Measurements support the visual impression that scrapie fibrils are particularly well endowed with EDs. For the fibrils shown in Fig. 12, the volume of EDs compared to the volume of protein density is 13.5% for a22L mouse scrapie (PrP fibril, single protofilament, EMDB 28089, PDB 8efu^7^), 8.5% for GSS (PrP fibril, paired protofilaments, EMDB 26613, PDB 7un5^28^), 9.0% for AD (tau PHF fibril, paired protofilaments, EMDB 26663, PDB 7upe^34^) and 4.3% for MSA (alpha-synuclein fibril, paired protofilaments, EMDB 10650, PDB 6xyo^17^). This extra bulk of EDs could explain the enhanced transmissibility of scrapie, if (as proposed here) it represents replicating RNA. The particular RNA sequence, including mooted palindromicity^27^, may also be important.

### The scrapie agent

Cryo-EM maps of fibrils from brains of mice and hamsters with five infectious scrapie strains were examined in the present study. As noted by their primary authors^4–8^, these maps show extra densities containing a second (unknown) component, other than protein. In the present study, a Y-shaped polymer consistent with RNA was found. In places, this formed a single chain, consistent with single-stranded RNA, a known cofactor in scrapie fibril formation^3^. However, at one location, there were two antiparallel chains forming a duplex, raising the intriguing possibility of replicative behaviour.

A limitation of the current work is that it examines a static picture (the mature fibril) in a potentially changing landscape. Thus, cofactors involved in fibril growth might be interchangeable during the natural history of the fibril, and the mature fibrils only offer a snapshot in time of the dynamic processes involved. This idea has effectively been alluded to already, above, in terms of the of the use of PTA in fibril preparation by one of the labs, where the PTA might have displaced native ligands.

By the same token, natural cofactors might alternate and exchange during fibril growth. Whereas it has been claimed that the sole use of phosphatidylethanolamine as cofactor in the generation of infectious prions in vitro is definitive evidence that prions are neither viruses or virinos^3,35^, the idea of cofactor exchange allows for a later switch to RNA.

The conclusion here that the ligand is likely RNA in scrapie fibrils is supported by comparison with an in vitro model from the Eisenberg lab^15^. In this, RNA was used to promote tau fibril formation and the resulting cryo-EM map shows RNA hydrogen bonded to protein at arginines 406 and histidines 407. The RNA in this model has a novel straight structure with a repeat distance of about 4.8 Å matching that of protein (Fig. 5b). Appearances suggestive of RNA in the Rocky Mountain scrapie fibrils are comparable with the RNA-induced tau fibrils from the Eisenberg lab. Furthermore, EDs, with morphologies consistent with RNA were a common feature of fibrils from multiple human neurodegenerations involving multiple proteins^11^, consistent with the idea that RNA is a natural ligand in fibrils from diverse neurodegenerations.

A further test is to consider whether the presence of RNA makes sense biologically. It is certainly well established that RNA promotes PrP^Sc^ amplification^3,25^ and becomes complexed to PrP^Sc^ in amyloid plaques in scrapie^23^. In such a role, it might be acting generically, similar to other cofactors such as phosphatidylethanolamine or heparan sulfate^3^. On the other hand, RNA is an information molecule, with possible ensuing advantages over other candidates.

It is worth remembering that all cofactors are not equal. For example, in experiments in vitro, poly(A) and poly(dT) were one hundred times more potent than poly(dA) in stimulating PrPres amplification, whilst synthetic poly(C) failed to stimulate PrPres amplification at all^25^. Such a pecking order suggests a competitive environment for potential ligands, and competition could be the basis for the proposed displacement of natural ligands by PTA, a very powerful polyanionic reagent. Furthermore, in the natural *milieu*, at different time points, various ligands might replace each other until a definitive ligand is in place.

Arguably, the specific base sequence of RNA exerts a specific tethering effect on the protein, resulting in the specific protein shape found in disease^11^. Thus specific neurodegenerative diseases might be associated with specific RNA sequences or strains.

It is also possible that specific sequences might have different neurotoxicities, since RNA toxicicity has been implicated as a factor in some neurodegenerations^36^.

In terms of neurotropism, the availability of particular RNAs in particular brain cells might selectively promote particular neurodegenerations (this idea can be seen as a version of the cofactor selection hypothesis of neurotropism^3^, applied here to different RNA sequences, rather than different chemical types of cofactors).

It is now established, on the basis of cryo-EM of ex vivo fibrils from diverse human neurodegenerations, that specific neurodegenerations are associated with specific protein conformations^32^. However, in in vitro studies, it is typically found that amyloids are polymorphic. This raises the possibility that fibrils ex vivo are subject to constraint, consistent with the effect of specific ligands, absent from the in vitro situation. In such a scenario, RNA, with its potential for sequence variation and template-driven synthesis, might have an advantage over other potential cofactors.

If RNA, an information molecule, is a component of the scrapie agent, could it engage in replication? The suggestion here of a duplex form of RNA, comprising two antiparallel chains, is consistent with but does not prove this possibility. The structure observed is different to double-stranded RNA but could feasibly be a refolded form of dsRNA. The rectilinear architecture of the fibril, with the protein and second component braced together by hydrogen bonds, might confer catalytic properties.

Whether or not RNA is an essential component of the scrapie agent remains moot. The evidence here does however lend to the possibility of a scrapie agent for which particular sequences of RNA might define strain and be capable of self-replication. It is important to note however, that such an agent would not be a conventional virus. In conventional viruses, nucleic acid and protein are colinear (i.e. the nucleic acid codes for the protein). In scrapie, the protein is coded for by the host DNA and not by any putative agent-associated RNA. Instead, as argued above, putative RNA in scrapie might be a short tandem repeat. Therefore, the scrapie agent might turn out to be neither a protein-only prion or a virus, but a completely new type of infectious agent.

## Methods

### Data sources and selection

Data for this study were sourced from public repositories, the EMDB^9^ and the PDB^10^. Cryo-EM maps and protein models of fibrils from brains of rodents with five scrapie strains were examined: 263K hamster^4^, RML mouse^5^, aRML mouse^6^, a22L mouse^7^ and ME7 mouse^8^. The protein involved in all cases is PrP. In two cases (aRML and a22L) the protein is anchorless. Three strains (263K, aRML and a22L) are from the Rocky Mountain (RM) lab and two (RML and ME7) are from University College London (UCL). This is an important distinction because phosphotungstic acid (PTA) was used by the UCL lab but not the RM lab in fibril preparation and has a bearing on the interpretation of EDs.

All fibrils had EDs, as determined by inspection and reference to the published papers. Lysine-coordinating EDs and arginine-coordinating EDs were the subject of the present study. EDs from other protein environments (e.g. those likely attributable to glycans and GPI anchors) were excluded. EDs were named by the EMDB number and the first coordinating residue. Observations were made on the following cryo-EM maps and atomic models: EMDB 23459 and PDB 7lna in 263K hamster scrapie^4^, EMDB 13989 and PDB 7qig in RML mouse scrapie^5^, EMDB 25824 and PDB 7tdt in aRML mouse scrapie^6^, EMDB 28089 and PDB 8efu in a22L mouse scrapie^7^ and EMDB 15043 and PDB 8a00 in ME7 mouse scrapie^8^. Mouse residue numberings are used in the current paper, unless otherwise specified. Observations were also made on human neurodegenerations for comparison, viz*.* EMDB 26607 and PDB 7umq and EMDB 26613 and PDB 7un5 in PrP fibrils in GSS^28^, EMDB 26663 and 7upe in tau PHF fibrils in AD^34^ and EMDB 10650 and PDB 6xyo in alpha-synuclein fibrils in MSA^17^.

The following were used in the section on candidate constituent molecules: EMDB 25364 and PDB 7sp1 in RNA-induced tau fibrils^15^, PubChem^37^ 90478052 glycogen from oyster, rebuilt as 1,4-linked polyglucose in UCSF ChimeraX^22^, PDB 3iri heparin^38^ and PDB 4l2h poly (ADP-ribose)^39^, extended in UCSF ChimeraX^22^.

### Observations and measurements

Observations and measurements were made in UCSF ChimeraX^22^ unless otherwise stated. A Dell Desktop-GUQ6HDT XPS 15 9500 with Intel® Core™ i7-10750H CPU @2.6 GHz with 16 GB RAM running Windows 11 Home (version 21H2) with broadband internet connection was used to run software and to access data and software online.

Each cryo-EM map was examined at the authors’ recommended contour level with the protein model in situ. For clarity, the map was made transparent and clipped to the region of the protein. EDs were identified in their protein environment, by inspection and reference to the published papers.

To determine whether EDs were in perfect register with the protein, markers were placed 30 units apart on EDs and 30 rungs apart on the adjacent protein density and the distances compared. To assess whether EDs were within hydrogen-bonding distance of protein, color zones from the NZ (terminal nitrogen) atoms of lysines were incremented until they reached the surface of the EDs. The angle between 3 markers at 10 unit intervals was measured as an index of straightness.

In some cases, EDs were detached from, or showed minor regions of fusion to, the protein density. In other cases, EDs were extensively fused to the protein density. EDs were isolated from the rest of the density, in order to observe and measure them freely. This was done with the map eraser tool, by erasing the ED and subtracting the remainder from a copy of the original. Where there was extensive fusion between the ED and the protein density, the ED was isolated by using the color zone and split map commands, at a radius of 2 Å from the protein atoms. Further detail was evinced by adjusting the contour level. For some depictions, the hide dust tool was used.

The molecular weight (MW) of the ED was calibrated to the MW of the protein. Protein density was isolated with the color zone and split map commands, with a 2 Å radius around protein atoms. Volumes of isolated EDs and protein densities were measured with the measure blobs tool or measure volume command. The MW of one rung of protein was calculated by opening the protein model in UCSF Chimera^40^ adding hydrogens, selecting one rung and invoking the keyboard shortcut ac mw. The ratio of the volume of ED to the volume of protein density was calculated. The ratio was multiplied by the MW of one rung of protein to give an estimate of the MW of one residue of ED. For comparison, the MW of RNA residues, in Daltons, are as follows: cytosine 304.2, uracil 305.2, adenine 328.2 and guanine 344.2 (average 320.5).

In order to create a molecular model of the protein component of the entire fibril, multiple copies of the PDB protein model were translated and fitted to the corresponding EMDB cryo-EM map using UCSF ChimeraX^22^. Electrostatic potential surfaces were created in UCSF ChimeraX^22^ using the coulombic command.

For comparing the structures of two strains, a script was run in UCSF ChimeraX^22^ in which the root mean square deviation (RMSD) of the alpha-carbon atoms of blocks of 11 residues were calculated sequentially and plotted against the central residue number. For example, the RMSD of residues 95 to 105 was plotted against residue number 100. The chart was annotated with the position of EDs and the variable regions were colour-coordinated with the atomic structures.

ED 28089-147 was split into two near-identical chains after it was observed to appear symmetrical. The ED, viewed at increased level (0.008), was split at a radius of 6 Å (determined empirically) from the terminal nitrogen atoms NH1 and NH2 of R147 in the corresponding protein model PDB 8efu^7^ using the color zone tool in UCSF ChimeraX^22^. The resulting chains were translated vertically in order to view them separately and then one fitted to the other using the fitmap command and the correlation metric noted from the log.

### Molecular docking

A 3mer of oRNA (UUU), modelled in silico as previously described^11^, was used as ligand. It was chosen with an inter-residue distance as close as possible to the rung distance of the protein. Five rungs of protein (cut down to coordinating and intervening residues) were used as receptor. Ligand and receptor were converted to PDBQT format in AutoDock Tools^41^. Both ligand and receptor were docked as rigid objects. Molecular docking was done with AutoDock Vina^20^. The resulting poses were examined in UCSF ChimeraX^22^. They were assessed for overlap with the ED, symmetrical hydrogen bonds (displayed with relaxed criteria, distance tolerance 0.4 A, angle tolerance 20°) and favourable AutoDock Vina score (affinity, kcal/mol). The protein and selected pose were combined in UCSF Chimera^40^ and submitted to the MolProbity^21^ Webserver to determine the clashscore. The duplex form of oRNA was modelled and docked as previously described^11^.

### Ethical statement

The cryo-EM maps and atomic models used in the present study were sourced from the EMDB and PDB public repositories from the publications recorded herein, which for patient data all affirm that ethical review and informed consent were obtained (all patient data for the present study was at one remove and no new patient data was obtained) and for animal data all affirm that appropriate licences were approved and granted (all animal data for the present study was at one remove and no new animal data was obtained).

## Data Availability

The EMDB and PDB accession numbers for data examined in this paper are provided in the text. Any other relevant data are available from the corresponding author upon request.
